# Validation of prediction models of severe disease course and non-achievement of remission in juvenile idiopathic arthritis part 2: results of the Nordic model in the Canadian cohort

**DOI:** 10.1186/s13075-019-2091-8

**Published:** 2020-01-15

**Authors:** Andrew Henrey, Veronika Rypdal, Martin Rypdal, Thomas Loughin, Ellen Nordal, Jaime Guzman, for the ReACCh-Out and NoSPeR Investigators, Roxana Bolaria, David A. Cabral, Mercedes Chan, Katherine Gross, Kristin Houghton, Kimberly Morishita, Stuart E. Turvey, Lori B. Tucker, Ross E. Petty, Susanne M. Benseler, Nicole Johnson, Nadia Luca, Paivi Miettunen, Heinrike Schmeling, Janet Ellsworth, Dax G. Rumsey, Kerstin Gerhold, Kiem Oen, Alan M. Rosenberg, Roberta A. Berard, Maggie Larché, Bonnie Cameron, Brian M. Feldman, Debbie Feldman, Ronald M. Laxer, Deborah M. Levy, Rayfel Schneider, Earl Silverman, Lynn Spiegel, Shirley M. Tse, Rae S. M. Yeung, Ciaran M. Duffy, Michele Gibbon, Roman Jurencak, Johannes Roth, Karen Watanabe Duffy, Anne-Laure Chetaille, Jean Dorval, Sarah Campillo, Claire LeBlanc, Gaëlle Chédeville, Rosie Scuccimarri, Julie Barsalou, Elie Haddad, Claire St. Cyr, Gilles Boire, Alessandra Bruns, Adam M. Huber, Bianca Lang, Suzanne E. Ramsey, Elizabeth Stringer, Paul Dancey, Natalie J. Shiff

**Affiliations:** 1 Department of Statistics and Actuarial Sciences, Simon Fraser University, Burnaby, British Columbia Canada; 2 Department of Pediatrics, University Hospital of North Norway, Tromsø, Norway; 3 Department of Clinical Medicine, UiT – The Arctic University of Norway, Tromsø, Norway; 4 Department of Mathematics and Statistics, UiT – The Arctic University of Norway, Tromsø, Norway; 5 Division of Pediatric Rheumatology, British Columbia Children’s Hospital, 4500 Oak Street, Suite K4-122, Vancouver, British Columbia V6H 3N1 Canada; 6 Department of Pediatrics, University of British Columbia, 4500 Oak Street, Suite K4-122, Vancouver, British Columbia V6H 3N1 Canada

**Keywords:** Juvenile idiopathic arthritis, Prediction model, Validation, Prognosis

## Abstract

**Background:**

Validated clinical prediction models to identify children with poor prognosis at the time of juvenile idiopathic arthritis (JIA) diagnosis would be very helpful for tailoring treatments, and avoiding under- or over-treatment. Our objective was to externally validate Nordic clinical prediction models in Canadian patients with JIA.

**Methods:**

We used data from 513 subjects at the 3-year follow-up from the Research in Arthritis in Canadian Children emphasizing Outcomes (ReACCh-Out) cohort. The predicted outcomes were non-achievement of remission, severe disease course, and functional disability. The Nordic models were evaluated exactly as published and after fine-tuning the logistic regression coefficients using multiple data splits of the Canadian cohort. Missing data was handled with multiple imputation, and prediction ability was assessed with C-indices. C-index values > 0.7 were deemed to reflect helpful prediction.

**Results:**

Overall, 81% of evaluable patients did not achieve remission off medications, 15% experienced a severe disease course, and 38% reported disability (CHAQ score > 0). The Nordic model for predicting non-achievement of remission had a C-index of 0.68 (95% CI 0.62–0.74), and 0.74 (0.67–0.80) after fine-tuning. For prediction of severe disease course, it had a C-index of 0.69 (0.61–0.78), and 0.79 (0.68–0.91) after fine-tuning. The fine-tuned Nordic model identified 85% of the cohort as low risk for a severe disease course (< 20% chance) and 7% as high risk (> 60% chance). The Nordic model to predict functional disability had a C-index of 0.57 (0.50–0.63), and 0.51 (0.39–0.63) after fine-tuning.

**Conclusions:**

Fine-tuned Nordic models, combining active joint count, physician global assessment of disease activity, morning stiffness, and ankle involvement, predicted well non-achievement of remission and severe disease course in Canadian patients with JIA. The Nordic model for predicting disability could not predict functional disability in Canadian patients.

## Background

Juvenile idiopathic arthritis (JIA) is a heterogeneous group of conditions characterized by chronic arthritis of unknown cause with onset before the age of 16 years [1]. Validated clinical prediction models to identify children with poor prognosis at diagnosis would be very helpful for tailoring aggressive treatments, such as synthetic and/or biologic DMARDS prescribed shortly after diagnosis, to patients with poor prognosis and prevent under- or over-treatment.

Clinical prediction models are relatively recent developments in JIA, but they are widely used to tailor treatments in practice guidelines, e.g., in cardiovascular disease [2] or osteoporosis [3]. Good practices for development of clinical prediction models and consensus statements for reporting these studies are available [4, 5]. Their discrimination accuracy is often assessed with the C-index, equivalent to the area under the Receiver Operating Characteristic curve (AUC), where 1.0 reflects perfect prediction and 0.5 reflects chance alone. In the cardiovascular literature, prediction models with C-index values > 0.7 are considered helpful and those with values > 0.8 are considered excellent [6].

Using data from the Research in Arthritis in Canadian Children Emphasizing Outcomes (ReACCh-Out) Cohort, Guzman et al. developed a clinical prediction model to predict a severe disease course that had a C-index of 0.85 in internal validation in that cohort [7]. Using data from the Nordic Study Group of Pediatric Rheumatology (NoSPeR) cohort, Rypdal et al. developed models to predict non-achievement of remission, functional disability, and articular damage 8 years after disease onset. For prediction of non-achievement of remission and functional disability, the C-indices in split validation sets were 0.78 and 0.73, respectively [8]. The mathematical models for Canadian and Nordic prediction tools are shown in Table 1, and user-friendly online calculators are available at https://shiny.rcg.sfu.ca/jia-sdcc/ and http://predictions.no.

**Table 1:** The original Canadian and Nordic prediction models for juvenile idiopathic arthritis

Source	Outcome predicted	Calculate *A*	Use *A* to calculate chance of outcome (%)
Guzman et al., Canada 2017 [7]	Severe disease course, defined by trajectory of quality of life, pain, active joint count, medication requirements, and medication side effects over the 5 years after diagnosis	*A* = − 2.92 + 0.18 × (active joint count at baseline) − 1.23 × (psoriatic arthritis) − 1.14 × (oligoarthritis) − 0.49 × (RF-negative polyarthritis) + 0.75 × (upper limb joint involvement) − 0.88 × (symmetric joint involvement) + 1.31 × (RF positivity) − 1.42 × (subtalar joint involvement) − 0.31 × (finger joint involvement) + 0.84 × (cervical spine involvement) + 0.48 × (ankle joint involvement) + 0.56 × (presence of morning stiffness) + 0.06 × (hip involvement) + 1.50 × (temporal mandibular joint involvement) + 0.54 × (mid-foot involvement) + 0.86 × (presence of enthesitis)	[*e*^*A*^/(1 + *e*^*A*^)] × 100 where *e*^*A*^ is the natural antilogarithm of *A*
Rypdal et al., Norway 2018 [8]	Non-achievement of remission 8 years after onset	*A* = − 1.58 + 0.04 × (cumulative joint count within 6 months of onset) + 0.03 × (ESR in mm/h) − 0.07 × (CRP > 10 mg/L) + 1.16 × (morning stiffness > 15 min) + 0.16 × (physician global assessment) + 1.25 × (ANA positive) + 1.37 × (B27 positive) + 1.10 × (ankle joint arthritis)	[*e*^*A*^/(1 + *e*^*A*^)] × 100 where *e*^*A*^ is the natural antilogarithm of *A*
Rypdal et al., Norway 2018 [8]	Functional disability (CHAQ > 0) 8 years after onset	*A* = − 1.68 − 0.02 × (cumulative joint count within 6 months of onset) + 0.01 × (ESR in mm/h) − 0.20 × (CRP > 10 mg/L) + 1.03 × (morning stiffness > 15 min) − 0.40 × (physician global assessment VAS) + 1.21 × (finger joint arthritis) + 0.77 × (pain VAS)	[*e*^*A*^/(1 + *e*^*A*^)] × 100 where *e*^*A*^ is the natural antilogarithm of *A*

*RF* rheumatoid factor, *ESR* erythrocyte sedimentation rate, *CRP* C-reactive protein, *ANA* antinuclear antibody test, *B27* human leucocyte antigen B27, *VAS* visual analogue scale from 0 to 10 with 10 indicating worse values

Although they aimed to predict different outcomes, there are similarities between the Canadian model to predict a severe disease course and the Nordic model to predict non-achievement of remission. Both are multivariable logistic regression models that combine routine clinical and laboratory variables available early in the disease and both include the active joint count, ankle involvement, and presence of morning stiffness. The main differences are that the Canadian model uses twice as many variables (16 vs 8), including JIA category, presence of enthesitis, and involvement of joints other than the ankles, and that the Canadian model uses active joint count at presentation, while the Nordic model uses cumulative joint count 6 months after onset.

External validation of clinical prediction models in populations different than those in which they were developed is essential before general adoption can be recommended [5]. The goal of this collaboration between ReACCh-Out and NoSPeR researchers was to determine if clinical prediction models developed in one cohort could be externally validated in the other cohort. The aim of the present study was to externally validate the Nordic models in Canadian patients. A twin study by Rypdal et al. externally validated the Canadian model in Nordic patients [9].

## Patients and methods

The ReACCh-Out cohort has been previously described in detail [10, 11]. In brief, 1497 patients newly diagnosed with JIA were recruited at 16 pediatric rheumatology centers across Canada from January 2005 to December 2010. The first visit occurred as soon as possible after diagnosis, but the time from diagnosis to the first visit could be as long as 1 year. Follow-up visits were scheduled every 6 months for 2 years and then yearly up to 5 years, or until May 2012. At each official study visit, full clinical information was collected, including the American College of Rheumatology (ACR) core variables [12], treatment information, and patient-reported outcomes. Erythrocyte sedimentation rate (ESR) and C-reactive protein (CRP) levels were only measured if clinically indicated. At interim clinic visits between study visits, a reduced dataset was collected, including the number of active joints, limited joints or enthesitis sites, treatment information, and ESR and CRP levels if measured. ReACCh-Out was approved by Research Ethics Boards at all participating institutions and performed in accordance with the Declaration of Helsinki, including informed written consent.

The Nordic Cohort recruited 500 patients newly diagnosed with JIA in defined geographical locations of Norway, Sweden, Finland, and Denmark in 1997–2000. First visit occurred approximately 6 months after disease onset, then at 12 months, and then every 1–3 years with an obligatory visit at approximately 8 years after disease onset (available for 440 subjects) [13].

### Patients

For the current study, the goal was to select patients recruited in ReACCh-Out who were as similar as possible to the population used for development of the original Nordic prediction models. We considered including only patients with information at the 5-year follow-up, but this would have reduced our sample size considerably. Moreover, since ReACCh-Out did not follow patients into adulthood, many children who entered the cohort as teenagers would have been excluded, resulting in under-representation of JIA categories commonly seen in teenagers. We chose instead to include data of patients recruited within 3 months of diagnosis who had enough information at the 3-year visit to ascertain the outcomes of interest.

### Outcomes

Our primary outcome was non-achievement of remission at the 3-year visit. We were not able to use the exact same outcome definition as in the original Nordic study, since the schedule of visits and other features differed between the two cohorts. We designated a primary definition and examined several alternative definitions. The primary definition of remission was clinical inactive disease for at least 12 months while off treatment [14]. We also examined the model’s ability to predict a severe disease course as defined by Guzman et al. [7], based on cluster analysis of changes in pain, health related quality of life, number of active joints, medication requirements, and medication side effects over 5 years.

Clinical inactive disease was defined as no active joints, no active extra-articular manifestations (no enthesitis, uveitis, or systemic manifestations), and a physician global assessment of disease activity (PGA) of < 1 cm in a 10-cm visual analogue scale (VAS). This definition was based on the 2004 Wallace criteria [14] and has been previously used by our group [11, 15]. The main differences relative to the current American College of Rheumatology (ACR) provisional criteria [16] are that a morning stiffness of 15 min or less and normal acute phase reactants were not required.

We defined functional disability as a Childhood Health Assessment Questionnaire (CHAQ) disability index [17] greater than 0 at the 3-year visit. This is the same instrument and cutoff used in the Nordic study, but at a different follow-up time. The Nordic study also developed a model to predict functional disability defined by the Child Health Questionnaire physical summary score [18], but the Canadian cohort did not use that instrument.

### Model validation

For each subject in the Canadian cohort, we first computed the probabilities of non-achievement of remission and functional disability, using the Nordic models exactly as published (i.e., with the same intercept and coefficients). We compared this prediction to the observed outcome to assess prediction accuracy (C-index and confidence intervals, details below). If the resulting value was substantially lower than the value originally published in the Nordic cohort, we proceeded to fine-tune the models. Fine-tuning means re-estimation of the model’s intercept and coefficients to better fit a new population, while keeping the same predictors and same logistic regression methods to combine predictors. Intercept and coefficients were re-estimated using multiple splits of the Canadian cohort.

In pre-specified sensitivity analyses, we assessed the ability of the Nordic model to predict alternative definitions of remission, including inactive disease while off treatment (i.e., without requiring 12 months) and inactive disease for > 6 months irrespective of treatment. We also looked at the model’s ability to predict a severe disease course, as defined by Guzman et al [7]. This analysis was not pre-specified. Similar to what was reported in the Nordic cohort [8], we looked at the performance of prediction models that excluded the laboratory variables from the prediction model. Additional post hoc analyses assessed the models’ performance after excluding patients with systemic JIA and in a subsample of patients who attended the 5-year follow-up. Lastly, we examined the prediction ability of a model that included only the active joint count at baseline.

### Statistical analysis

All analyses were conducted using R software. The Canadian cohort had an overall 10% missing rate of baseline data. Missing data were imputed in 20 datasets using the method of multiple imputation by chained equations (MICE) [19]. Outcome data was not imputed. Our reported average C-indices and average coefficient estimates are unweighted means across all 20 imputed datasets. We followed Rubin’s rules [20] to compute standard errors (SEs) for all quantities across the 20 imputed datasets.

To validate the original un-tuned Nordic models in Canadian children, we fit each model to 100% of the data within each of 20 imputed datasets. From each dataset, we computed the C-index and the SE of the C-index. We then combined these individual SEs to produce the overall C-index SE.

For the fine-tuned models, we needed to ensure that the model-evaluation statistics were computed on data not used to estimate the coefficients. We followed the procedure published by Jiang et al. [21] and modified it to compute the C-index. For a given imputed dataset, we estimated the average C-index using their recommendation of the Leave-One-Out Cross-Validation (LOOCV) error. To estimate the within-dataset standard error, we used their recommendation of a nested cross-validation within a bootstrap (the BCCV algorithm). We created *B* = 25 bootstrap samples on an imputed dataset. Within each bootstrap sample, we removed one original observation (if it occurred multiple times in the imputed data, we removed all cases) and predicted this observation using the fitted model. We repeated this process for each observation in turn to obtain predictions on each case. We then computed a C-index on all predicted values of that bootstrap sample. We then computed the standard deviation (SD) of the *B* = 25 bootstrap sample C-indices as an estimate of the within-dataset SD of the C-index. The between-dataset and within-dataset SDs were combined to produce the overall multiple imputation SE using Rubin’s rules [20].

To obtain SE of coefficients, we fitted the model on each of *B* = 25 bootstrap samples from each imputed dataset (a total of 500 fits). For each imputed dataset, we estimated the within-dataset SE of the coefficients using the SD of the coefficient estimates from the glm package in R across the 25 bootstrap samples. Again, we combined this with the between-dataset SD to get the overall SE.

## Results

**Fig. 1:**
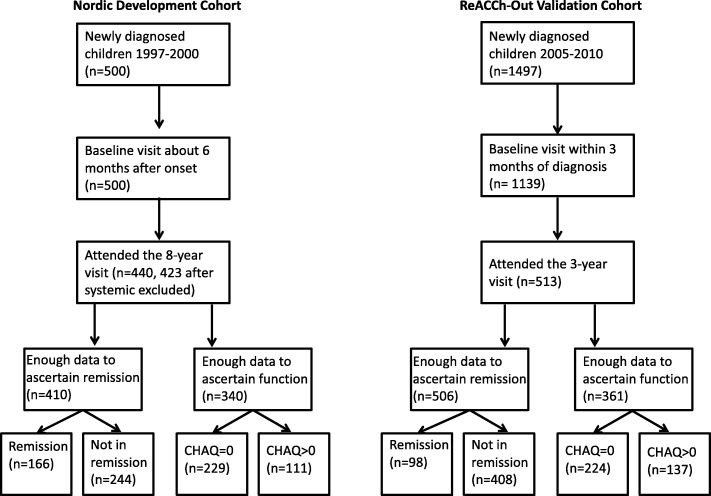
Patient flow charts for the development and validation cohorts

A total of 513 subjects fulfilled our inclusion criteria at the 3-year visit, which occurred on average 3.75 years after JIA onset. The patient flow chart is shown in Fig. 1. The figure also shows the corresponding patient flow chart used to select subjects for the original Nordic study.

Baseline patient characteristics in the validation cohort are compared with the reported characteristics in the original development cohort in Table 2. Overall, the cohorts are similar to each other and to other inception cohorts of JIA reported in western populations. The original Nordic study excluded patients with systemic JIA from model development and had only four patients with RF-positive polyarthritis [8].

**Table 2:** Baseline characteristics for patients in the development and validation cohorts according to non-achievement of remission

q	Nordic development cohort (*N* = 427)		ReACCh-Out validation cohort (*N* = 506)	
	Remission (*n* = 181)	Non-remission (*n* = 246)	Remission (*n* = 98)	Non-remission (*n* = 408)
Characteristics				
Age at onset, years	5.9 (2.5, 10.0)*	5.2 (2.5, 9.5)	8.0 (3.6, 11.5)	7.2 (2.6, 11.1)
Female, *n* (%)	115 (63.5)	169 (68.7)	62 (63.3)	285 (70.4)
Onset to enrolment, months	7 (6, 8)	6.5 (6, 8)	3.9 (2.4, 6.0)	5.1 (2.7, 9.7)
JIA category, *n* (%)**				
Oligoarthritis	107 (59.1)	111 (45.1)	55 (56.1)	137 (33.6)
RF-neg. polyarthritis	25 (13.8)	68 (27.6)	4 (4.1)	113 (27.7)
RF-pos. polyarthritis	1 (0.6)	3 (1.2)	0 (0.0)	17 (4.2)
Systemic	15 (8.3)	2 (0.8)	13 (13.3)	28 (6.9)
Enthesitis-related	11 (6.1)	23 (9.3)	8 (8.2)	50 (12.3)
Psoriatic	3 (1.7)	3 (1.2)	8 (8.2)	20 (4.9)
Undifferentiated	19 (10.5)	36 (14.6)	10 (10.2)	43 (10.5)
Assessments and laboratory tests				
Active joints, *n* (%)				
Cervical arthritis	13 (7.2)	25 (10.2)	0 (0.0)	26 (7.0)
Finger arthritis	40 (22.1)	94 (38.2)	16 (19.3)	167 (44.7)
Ankle arthritis	65 (35.9)	129 (52.4)	17 (20.5)	186 (49.7)
Hip arthritis	24 (13.3)	38 (15.4)	7 (8.4)	50 (13.4)
Active joint count***	2 (1, 4)	4 (2, 7)	1 (1, 2)	3 (1, 9)
Physician global assessment	0.8 (0.0, 1.3)	2.0 (1.0, 3.8)	1.9 (1.0, 3.2)	3.8 (2.0, 6.0)
Parent global assessment	0.6 (0.0, 2.0)	1.7 (0.5, 3.5)	0.7 (0.2, 2.4)	2.3 (0.7, 4.9)
Pain	0.4 (0.0, 3.0)	2.3 (0.5, 4.2)	1.0 (0.2, 3.0)	3.9 (1.0, 6.1)
CHAQ DI	0.1 (0.0, 0.6)	0.5 (0.0, 1.1)	0.1 (0.0, 0.5)	0.5 (0.1, 1.1)
Stiffness > 15 min (%)	30 (16.6)	90 (36.6)	45 (55.6)	184 (65.0)
ESR****	11.5 (6, 20)	17.5 (10, 31)	20 (9, 34)	21 (9, 40)
CRP****	0.0 (0, 0)	0.0 (0, 15)	2.0 (0.2, 10)	3.0 (0.3, 17)
ANA	37 (20.4)	76 (30.9)	49 (50.0)	182 (50.7)
RF	5 (2.8)	5 (2.0)	5 (5.1)	23 (6.4)
HLA B27	22 (12.2)	60 (24.4)	5 (5.1)	36 (10.0)
Treatment by first study visit (%)				
NSAIDs	152 (84.0)	215 (87.4)	88 (89.8)	390 (95.6)
Joint injections	84 (46.4)	152 (61.8)	24 (24.5)	88 (21.6)
DMARDs	20 (11.0)	71 (28.9)	9 (9.2)	137 (33.6)
Biologics	0	0	1 (1)	1 (0.2)

*Numbers are median (25th centile, 75th centile) or number of patients (%)

**Patients with systemic JIA were excluded from the Nordic prediction model development study. They are included in the validation cohort and in this table

***The Nordic development cohort used the cumulative active joint count within 6 months of disease onset, and the ReACCh-Out validation cohort used the active joint count at baseline

****Erythrocyte sedimentation rate measurements were available for 322 of 427 patients (75.4%) in the Nordic cohort and for 458 of 506 patients (90.5%) in the ReACCh-Out cohort. C-reactive protein measurements were available for 345 of 427 patients (80.8%) in the Nordic cohort and 404 of 506 patients (79.8%) in the ReACCh-Out cohort

**Fig. 2:**
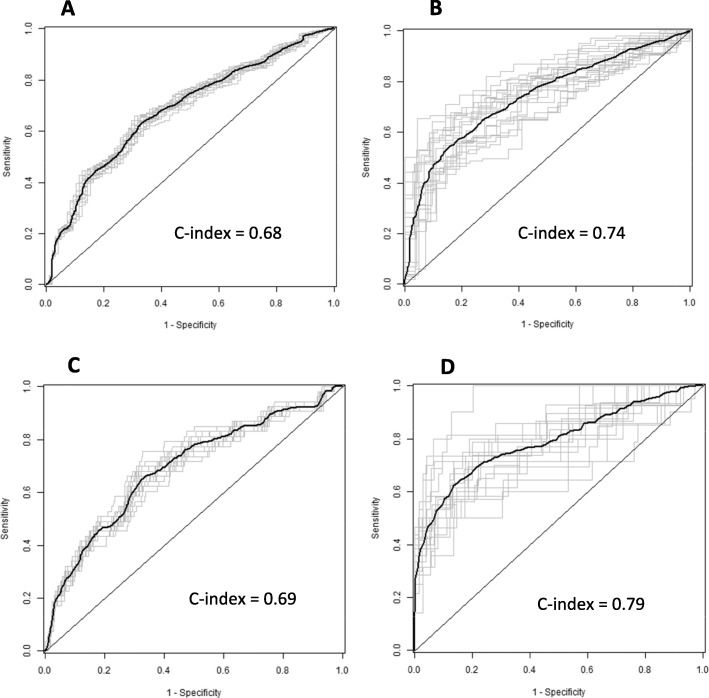
Receiver Operating Characteristic (ROC) curves for the Nordic model to predict non-achievement of remission when applied to Canadian data. **a** Original model predicting non-remission. **b** Fine-tuned model predicting non-remission. **c** Original model predicting a severe disease course. **d** Fine-tuned model predicting a severe disease course

In total, 408 of 506 evaluable Canadian patients (81%) were not in remission at the 3-year visit. Applying the Nordic model for prediction of non-achievement of remission exactly as published resulted on a C-index of 0.68 (95% CI 0.62–0.74). As this was lower than the published value (median AUC 0.78, IQR 0.72, 0.82), we proceeded with fine-tuning of coefficients. After fine-tuning, the C-index tested in multiple splits of the Canadian cohort was 0.74 (0.67–0.80). Figure 2 shows the corresponding Receiver Operating Characteristic (ROC) curves (panels a and b). The coefficients for original and fine-tuned models are shown in Table 3. Excluding patients with systemic JIA had a small impact on model performance, with a C-index of 0.73 (0.66–0.80) for the original model and 0.76 (0.69–0.83) for the fine-tuned model.

**Table 3:** Changes to model coefficients for the Nordic model to predict non-achievement of remission made during the fine-tuning process

Variable	Original Nordic	Fine-tuned Canada to predict non-achievement of remission		Fine-tuned Canada to predict severe disease course	
		With lab tests	No lab tests	With lab tests	No lab tests
Constant (intercept)	- 1.58 (- 0.70, -2.46)*	0.24	0.17	− 2.9	− 2.8
Active joint count**	0.04 (- 0.06, 0.14)	0.16	0.15	0.22	0.21
ESR in mm/h	0.03 (- 0.01, 0.07)	- 0.01	–	− 0.01	–
CRP > 10 mg/L	- 0.07 (- 1.45, 1.31)	0.12	–	0.08	–
Morning stiffness > 15 min	1.16 (0.26, 2.06)	0.42	0.38	0.23	− 0.03
Physician global assessment	0.16 (- 0.76, 1.08)	0.15	0.14	− 0.05	− 0.06
ANA positive	1.25 (0.25, 2.25)	0.03	–	− 0.56	–
HLA-B27 positive	1.37 (0.29, 2.45)	1.07	–	0.85	–
Ankle joint arthritis	1.10 (0.12, 2.08)	0.52	0.53	− 0.70	− 0.70
C-index (95% CI)	0.68 (0.62, 0.74)	0.74 (0.67, 0.80)	0.74 (0.67, 0.81)	0.79 (0.68, 0.91)	0.79 (0.69, 0.89)

*Numbers in parentheses are the 95% confidence interval

**The Nordic cohort used the cumulative active joint count within 6 months of disease onset, while the ReACCh-Out cohort used the active joint count at baseline

In secondary analyses, the C-index values calculated when using alternative definitions of remission were nominally lower than when using our primary definition of remission. For inactive disease while off treatment, it was 0.66 (0.60–0.71), and after fine-tuning, it was 0.69 (0.63–0.75). For inactive disease > 6 months irrespective of treatment, it was 0.62 (0.53–0.71), and after fine-tuning, it was 0.63 (0.50–0.75). We also calculated the C-index for a subsample of patients assessed at the 5-year follow-up in the ReACCh-Out cohort; the C-index was 0.57 (0.35–0.79), but this subsample was no longer representative of all patients with JIA since patients diagnosed as teenagers were not followed into adulthood, and the subsample was small, resulting in wide confidence intervals.

**Fig. 3:**
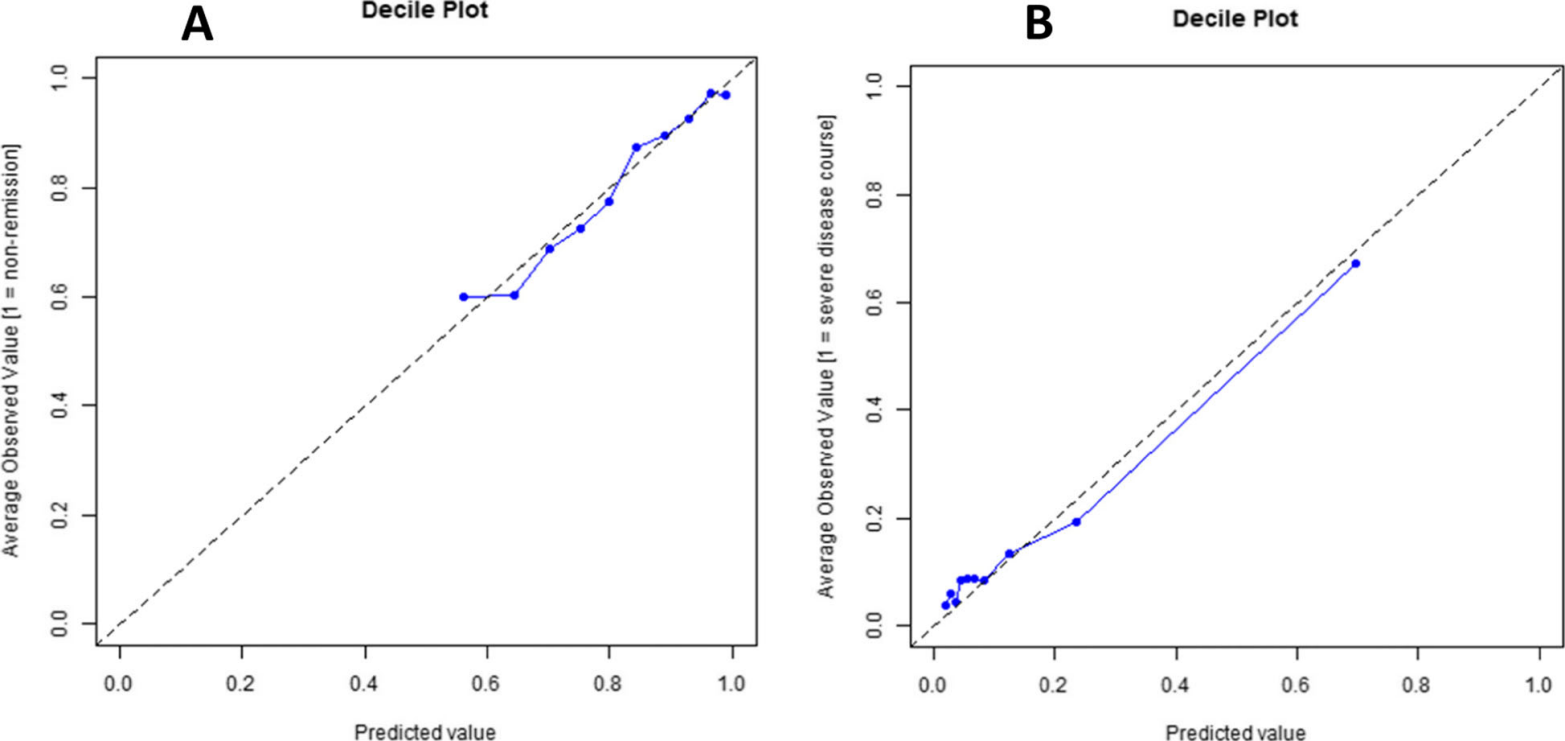
Calibration curves for the Nordic model to predict non-achievement of remission fine-tuned to Canadian data. **a** When predicting non-achievement of remission. **b** When predicting a severe disease course. Each point represents one tenth of the testing patient sample, arranged from lowest to highest probability of the outcome

A severe disease course was observed in 53 of 354 (15%) evaluable patients. Prediction with the Nordic model had a C-index of 0.69 (CI 0.61–0.78), and after fine-tuning, it was 0.79 (0.68–0.91). The corresponding ROC curves are shown in Fig. 2c, d. The calibration curves for the fine-tuned Nordic models are shown in Fig. 3. The Nordic model fine-tuned for severe disease course identified 85% of the cohort as low risk for severe disease (< 20% chance) and 7% of the cohort as high risk (> 60% chance).

We also examined the predictive ability of the model after exclusion of laboratory variables as it was done in the original publication. Fine-tuned versions with no laboratory values had a C-index of 0.74 (CI 0.67–0.81) when predicting non-achievement of remission and 0.79 (CI 0.69–0.89) when predicting a severe disease course, virtually the same values as models including laboratory variables. Lastly, a model using the baseline active joint count alone had a C-index of 0.66 (0.61–0.71) to predict non-achievement of remission and 0.76 (0.66–0.86) to predict a severe disease course.

Functional disability defined as a CHAQ > 0 was reported by 137 of 361 (38%) evaluable patients. Prediction with the Nordic model for functional disability had a C-index of 0.57 (0.50–0.63), and fine-tuning of coefficients was not able to improve accuracy, with a C-index of 0.51 (0.39–0.63). The corresponding ROC curves are shown in Additional file 1: Figure S1.

We note that the Nordic model for functional disability differed from the model for non-achievement of remission not only by the value of its coefficients, but also by the set of predictor variables. In the study of Rypdal et al., there was no model for prediction of severe disease course [8], and we used the model for non-achievement of remission when we tested for ability to predict severe disease course.

## Discussion

The aim of this study was to externally validate prediction models for poor prognosis in JIA developed in the Nordic cohort by assessing their performance in Canadian patients enrolled in the ReACCh-Out cohort. We found that after fine-tuning of coefficients, the Nordic model for predicting non-achievement of remission 8 years after disease onset had good accuracy to predict non-achievement of remission 3.75 years after onset (C-index 0.74) and a severe disease course over 5 years (C-index 0.79) in Canadian patients, even after laboratory variables were excluded. As shown in Table 3, fine-tuning of the model to predict non-achievement of remission increased the relative contribution of active joint count (beta coefficient changed from 0.04 to 0.16) and decreased the relative contribution of morning stiffness, ankle joint arthritis, and laboratory test results. The contribution of the physician global assessment was virtually the same (from 0.16 to 0.15). In contrast, the model to predict functional disability had a low C-index of 0.57 and fine-tuning did not improve accuracy (C-index 0.51).

For decades, prognostic research in JIA has concentrated on identifying features of poor prognosis [22], but the last decade has seen publication of several models that combine prognostic features to estimate the likelihood of an outcome for each patient. In 2012, Bulatovic et al. reported a model to predict non-response to methotrexate with an AUC of 0.65 [23], and in 2015, van Dijkhuizen et al. reported a model to predict methotrexate intolerance with C-index of 0.77 in internal validation [24]. More recently, van Dijkhuizen et al. combined clinical characteristics, Luminex biomarkers, and microbiota information to predict attainment of inactive disease within 2 years of diagnosis, but the resulting overall model was deemed not satisfactory with a AUC-like statistic of 0.65 [25]. Also recently, Guzman et al. used routine clinical and laboratory data at the time of diagnosis to predict early remission on medication (within 1 year of diagnosis) and the resulting model had a C-index of 0.69 in internal validation, just short of the conventional threshold of > 0.7 to consider a prediction model helpful [26].

In the context of these studies, our current findings raise four important questions: (1) Does the timing of outcome measurement influence our ability to predict inactive disease or remission? (2) Is the overall course of JIA a better prediction target than remission at a single point in time? (3) Should we eliminate laboratory values from the Nordic model altogether? (4) Is the fine-tuned Nordic model a better model to predict JIA disease course than the Canadian model?

In our opinion, the timing of assessment of inactive disease and remission will indeed influence the accuracy of a prediction model, particularly since it is well known that early in the course of JIA patients often transition in and out of inactive disease with subsequent visits [27, 28]. Later in the disease course, remission off medications may be a relatively stable target. This may be one reason why the Nordic model performed slightly better when predicting remission at 8 years in the original cohort than when predicting remission at 3.75 years in the current study. In addition to shorter follow-up, there were some differences in cohort composition, in ascertainment of predictors, and in the definition of inactive disease.

Whether the overall disease course is a better prediction target than remission is open to discussion. It is somewhat surprising that the Nordic model developed for predicting non-achievement of remission performed better at predicting a severe disease course than non-achievement of remission, since the severe-disease-course outcome is constructed very differently from non-achievement of remission. The results suggest that there are strong dependencies between outcome variables that are not fully understood, and that data-driven outcome measures, such as severe disease course, may be more valuable than previously assumed. The definition of a severe disease course is based on the overall trajectory of variables that are meaningful for families and clinicians, instead of accepted JIA core variables measured at a single point in time [7]. That said, remission is a well-accepted and easy to comprehend concept, although using ACR criteria for inactive disease [16] identifies a different patient population than using JADAS criteria [29, 30]. In the context of prediction studies, a targeted outcome needs to be useful for clinical decision-making but also well-suited for prediction. Future work should focus on rigorous clinical definitions of predicted outcomes. Such definitions will facilitate more accurate validation studies across cohorts.

It is remarkable that the exclusion of laboratory values (ESR, CRP, ANA, B27) had negligible impact on model accuracy, replicating the original findings in the Nordic cohort [8]. This means that a simple combination of active joint count, physician global assessment of disease activity, morning stiffness > 15 min, and presence of ankle involvement at baseline predicts well non-achievement of remission 3 or 8 years later, as well as a severe disease course during the first 5 years after diagnosis. Now that this has been demonstrated in both cohorts, it is hard to think of a good reason to keep laboratory values in the Nordic model.

The final question, which model is preferable, is also open to discussion. Although the Nordic model is simple and a simpler model is generally preferable, our results suggest that the accuracy of the fine-tuned Nordic model is somewhat lower than that of the Canadian model (C-index of 0.79 vs 0.85), but this could be simply due to the fact that the latter model was developed in the same Canadian cohort used in this study. A definitive answer to this question may require testing both models side by side in a third separate independent cohort.

### Study strengths and limitations

The main strength of our study is that it provides external validation of the Nordic prediction model in an entirely independent inception cohort with prospectively determined outcome measures. Study limitations include that our definition of remission is not exactly the same and the timeline for assessment is shorter than in the original study. A second limitation is the 10% rate of missing data on predictors, but we used multiple imputation by chained equations, which is a well-established method. A third limitation is that we used the baseline active joint count, instead of the cumulative active joint count within 6 months of disease onset used in the original Nordic model, yet we suspect they would be very similar, given that the baseline active joint count was obtained around the time of diagnosis and the start of treatment. Lastly, the observed improvements in accuracy with fine-tuning of coefficients suggest that for optimal accuracy, the Nordic model should be fine-tuned to the population in which it will be used. This may be problematic as the necessary cohorts for fine-tuning are only available in a few countries. Alternatively, this could indicate slight overfitting during model development in the Nordic cohort.

## Conclusions

The Nordic model developed to predict non-achievement of remission 8 years after JIA onset accurately predicted non-achievement of remission 3.75 years after onset and the overall disease course over 5 years after diagnosis in a Canadian cohort after the model coefficients were fine-tuned. The model is simple (active joint count, physician global assessment, morning stiffness, and ankle involvement with or without routine laboratory results), and it should be tested in clinical care to assess whether it improves the tailoring of treatment, i.e., more aggressive treatments for patients at high risk of non-achievement of remission, and whether this actually changes the subsequent disease course and prognosis. This should in turn lead to increased cost-effectiveness of care and, most importantly, improved patient outcomes.

## Supplementary information

**Additional file 1 : Figure S1.** Receiver Operating Characteristics (ROC) curves for the Nordic prediction model to predict disability

## Data Availability

Data is available to research teams that include at least one ReACCh-Out investigator and have a research protocol approved by the Scientific Protocol Evaluation Committee of the Canadian Alliance of Pediatric Rheumatology Investigators.

## Abbreviations

ACR: American College of Rheumatology
ANA: Antinuclear antibody test
AUC: Area under the Receiver Operating Characteristic curve
CHAQ: Childhood Health Assessment Questionnaire disability index
CI: Confidence interval
CRP: C-reactive protein
DMARDs: Disease modifying anti-rheumatic drugs
ESR: Erythrocyte sedimentation rate
HLA-B27: Human leucocyte antigen B27
IQR: Interquartile range, 25th, 75th centiles
JIA: Juvenile idiopathic arthritis
PGA: Physician global assessment of disease activity
NoSPeR: Nordic Study Group of Pediatric Rheumatology
NSAIDs: Non-steroidal anti-inflammatory drugs
ROC: Receiver Operating Characteristic curve
ReACCh-Out: Research in arthritis in Canadian children emphasizing outcomes
RF: Rheumatoid factor
SD: Standard deviation
SE: Standard error
VAS: Visual analogue scale
