# Supplementary material for: Validation of prediction models of severe disease course and non-achievement of remission in juvenile idiopathic arthritis part 2: results of the Nordic model in the Canadian cohort
Source: Arthritis Res Ther. 2020 Jan 15;22:10. doi: 10.1186/s13075-019-2091-8 (PMC6964007; doi:10.1186/s13075-019-2091-8)
Supplement: Supplementary file 1 — Additional file 1 : Figure S1. Receiver Operating Characteristics (ROC) curves for the Nordic prediction model to predict disability [file 13075_2019_2091_MOESM1_ESM.docx]

**Figure S1:** Receiver Operating Characteristic (ROC) curves for the Nordic prediction model to predict functional disability. A) Original model, B) Fine-tuned model


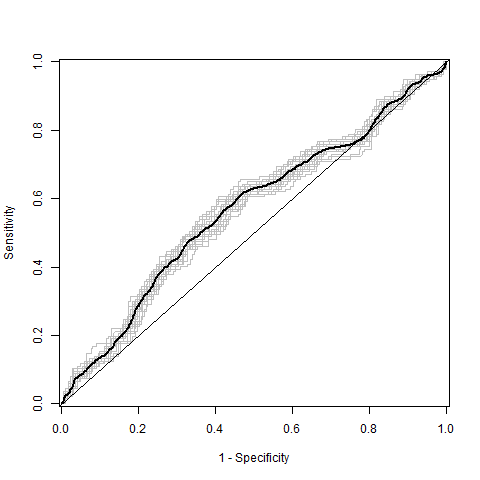

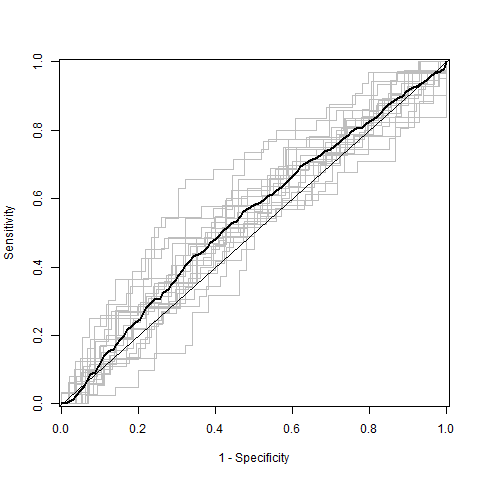


**B**

C-index = 0.51

C-index = 0.57

**A**
